# Surface Urban Heat Island Intensity and Urban Utility Consumption: Impact Analysis and Projections

**DOI:** 10.1155/tswj/3250112

**Published:** 2025-02-24

**Authors:** Gashaw T. Mekonnen, Arega B. Berlie, Mesfin A. Wubie, Solomon A. Legesse

**Affiliations:** ^1^Geography and Environmental Studies, Faculty of Social Sciences, Bahir Dar University, Bahir Dar, Amhara, Ethiopia; ^2^Geography and Environmental Studies, College of Social Sciences and Humanities, Dire Dawa University, Dire Dawa, Ethiopia; ^3^Natural Resource Management, College of Agriculture and Environmental Sciences, Bahir Dar University, Bahir Dar, Amhara, Ethiopia

**Keywords:** Amhara, electricity consumption, Gondar, SUHI, SUHII, water consumption

## Abstract

The rise in urban temperature has several impacts on the urban population. It manifests in water consumption, quality, and availability; energy consumption; greenhouse gas emissions; ecological disturbances; and human health. Studies have been conducted on the severity of the impact of surface urban heat island intensity (SUHII) on these variables at different scales in different parts of the world. The majority of the studies in Ethiopia considered the effect of per capita income on per capita water and energy consumption, disregarding the effect of temperature. However, this study tried to investigate the impact of SUHII on these utilities. It tested to see future trends in consumption in line with future SUHII patterns from 2024 to 2040. The present impacts were investigated using the path model and the future trends in consumption with autoregressive integrated moving average (ARIMA). The models' efficiency was checked using the sum of square error (SSE), mean square error (MSE), mean absolute percentage error (MAPE), root mean square error (RMSE), and archaic information criterion corrected (AICC), respectively. The best ARIMA models for SUHII, water, and electricity consumption were (3, 1, 1), (2, 1, 2), and (1, 1, 2) with AICC values of 13.72, −5.44, and 17.67, respectively. The result of the path model analysis buttressed that SUHII has a more significant impact on water (2.378 m^3^/1°C/annum) than electricity (1.616 kW/1°C/annum). The ARIMA model confirmed an increase in future water and electricity consumption. These results suggest that urban planners should consider the effects of SUHII on water and energy demand when they evaluate growth strategies and use incentives to encourage efficiency and sustainability.

## 1. Introduction

Urban heat island (UHI) has historically found expression in temperature excess over that in the rural environs [1]. This effect of UHI is central to urban climatology [2]. Given the large and ever-increasing number of urban inhabitants globally and the profound effects of cities and their inhabitants on the atmosphere, both within and beyond urban limits, ever-increasing attention is being directed to the study of the effects of urban climates [3]. The severity of the effect is often lessened as one moves away from the center of the city to the outskirts since building geometry in the central portion of the city is able to produce a more pronounced nocturnal temperature at the center than in the outskirts [4]. This is also ascertained by Unger, who states the impact of urban center geometry on heat distribution from the center to the periphery [5]. The variation in surface urban heat island (SUHI) in urban and rural areas is mainly due to variations in land use [6], building materials and heights [7], building geometry [5], and building spacing among other natural and artificial surfaces [8]. As a result, the density and pattern of urban development play a crucial role in determining the severity of the SUHI effect [2].

SUHI has an impact on human health and results in heat-related death [9]. According to Lucky, the implications of UHI include those on energy consumption, human health, and water quality and consumption [7]. The phenomenon amplifies summertime cooling energy demands in buildings [8] and also affects the productivity of the urban population [10]. Jabbar, Hamoodi, and Al-Hameedawi identify the impacts to be on human health and comfort [11]. Others also attached the impacts to elements of life such as morbidity, mortality, birth weight decrease, and social strife [12]. The increase in ambient temperature due to SUHI adversely impacts the cooling energy consumption of buildings and raises peak electricity demand [13]. It also raises the urbanites demand for water [14]. Therefore, analyzing the present impacts and projecting future impacts in line with SUHI is very vital to minimize the mentioned effects and for urban planning and policymaking [15]. When planning energy and water supply and demand, planners should weigh the effects on larger and smaller cities and urban and rural small towns since impacts are size-selective [16].

The primary goal in the planning and operation of water and electricity distribution systems is to meet the needs of consumers. This involves consistently delivering high-quality water at sufficient volumes and pressure, ensuring a dependable distribution system for both water and electricity. Effectively managing such a system requires accurate short-term forecasts for water and electricity usage [17]. A critical component of planning regional supply systems is understanding the current and future effects of surface urban heat island intensity (SUHII) on residential water and electricity consumption [15]. Analyzing the impact of water and electricity usage both now and in the future is increasingly important for the design, operation, and management of supply systems. This includes tasks like planning for new developments, expanding infrastructure, and estimating the needs of reservoirs, substations, pumping stations, and pipelines, as well as addressing urban management issues such as pricing and consumption restrictions [17]. According to Bougadis, Adamowski, and Diduch, short-term demand projections enable managers to make more informed decisions when balancing supply with residential demand. Estimating residential consumption volumes helps assess the additional needs for human use and the relationship between residential demand and the overall availability of electricity and water, as well as the demands of other sectors like industry and agriculture [18]. Moreover, analyzing the future impacts of water and electricity consumption is crucial for scheduling maintenance and making investment decisions, especially in future liberalized markets with fluctuating prices. Appropriate impact analysis and forecasting tools for the electricity and water consumption in Gondar have been used to address these needs.

The SUHII effect has spatial and temporal variations in and around cities where the core city is more highly affected by the daytime SUHI effect than the nighttime [19]. Empirical studies of SUHIs' effects, particularly those on human health [20], urban ozone concentration [21], energy use [22], and water use [23] in various nations, have been conducted as a result of increased awareness of their potential significance in urban environments. Studies linking the increase in water consumption [23] and electricity usage [10] with urban warming (heat island intensity) have been done in the United States in Phoenix (Arizona). Similarly, in Greece (Athens), the impact on energy consumption was investigated by Santamouris et al. [24].

Studies attempted to link the increase in per capita water and electricity consumption in Ethiopia to economic expansion and population increase [25], urbanization [26], and growth in per capita GDP and employment [27], ignoring the impact of temperature. However, prior research on the impact of SUHIs on current and future household energy and water use is lacking. It is anticipated that rising urban temperatures will have an impact on water and energy use by increasing demand and consumption. For a sustainable supply of water and energy to consumers with great quality, quantity, and pressure, it is crucial to analyze the impact and observe future consumption in line with the pattern of SUHII [28]. Urban utility consumption projections are necessary for effective planning [29] and sustainable management of these resources [30]. And it is also helpful in supporting decision-making processes and efficient financial use [14]. Therefore, this study investigates whether Gondar's SUHII has an impact on residents' per-capita energy and water use in the present and in the future.

## 2. Research Methodology

### 2.1. Study Area Description

#### 2.1.1. Physical Characteristics of Gondar City

**Figure 1:**
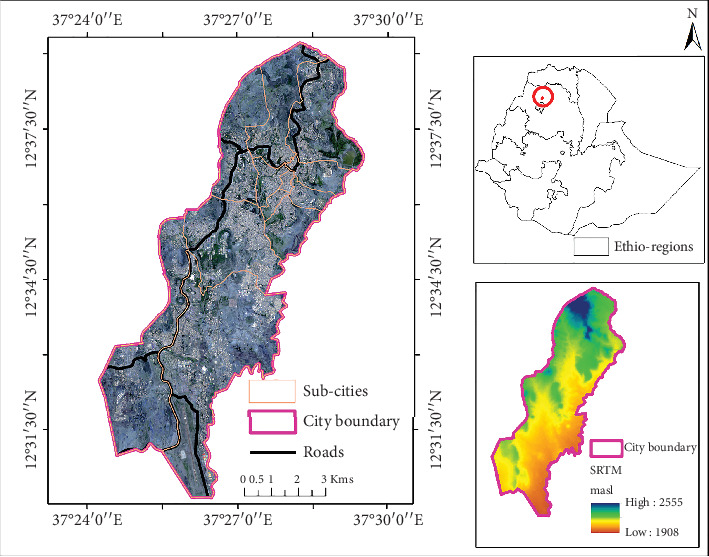
Map of the study area.

Gondar City, located 740 km from Addis Ababa (Ethiopia's capital) and 156 km from Bahir Dar (the capital of the Amhara region), is situated between 12°30⁣′ N to 12°39⁣′ N latitude and 37°24⁣′ E to 37°29⁣′ E longitude (Figure 1). As of 2018, the city covered an area of 72.6 km^2^. Surrounded by mountains, it is divided into 12 subdistricts, 25 urban kebele administrations, and 11 rural kebele administrations [31].

**Figure 2:**
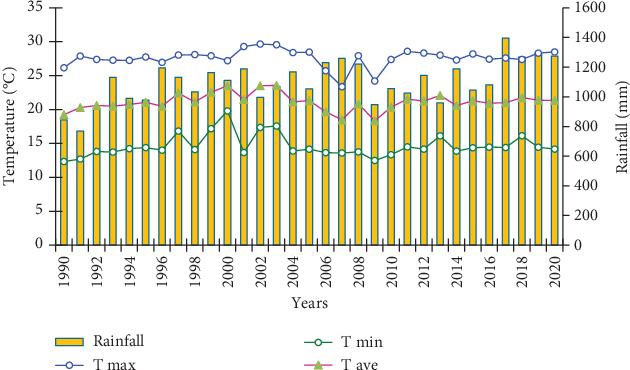
Temperature and rainfall graph of the study area (*source:* based on station data taken from the Ethiopian Meteorological Agency).

Figure 2 shows the temporal distribution of air temperature for the past 30 years. As it is indicated, the average temperature scaled up in 2000 and reached 24°C, and it dropped in 2007 and reached 18°C. The annual average maximum temperature is 27°C in 2002, and it was 22°C in 2007. The highest minimum temperature was observed in 2000, and it was 19°C, and the lowest average minimum temperature was recorded in 2001, and it was 11°C. The highest temperature is recorded during the spring and winter seasons since they are dry seasons where the area receives higher insolation. During summer and autumn, a relatively lower temperature is recorded since days and nights are colder, and the receipt of insolation is obstructed by dense cloud cover. In general, temperature is higher during the dry season and relatively lower during the rainy season.

Typically, in tropical areas, there are no distinct seasons. Areas included in this region have dry and wet times. But traditionally, Ethiopia is classified to have four agroecological zones experiencing the four seasons. The study area experiences four wet months and eight dry months. Summer is the season where the study area gets higher rainfall. During autumn, the receipt of rain begins to reduce. Winter and spring are dry seasons, but at the end of spring, particularly at the end of May, it begins to rain. Generally, as studies indicate, rainfall is variable but higher during the rainy seasons. Figure 2 indicates the temporal rainfall distribution of the study area in 30 years. The maximum rainfall is 1400 mm recorded in 2017, and the minimum is 800 mm recorded in 1991. Usually, maximum rain is recorded during summer since it is the rainy season in the study area.

#### 2.1.2. Socioeconomic Environment of Gondar City

Gondar's population grew significantly from 80,886 in 1984 to 207,044 in 2007. By 2022, the population had reached 395,138, making it the fifth-largest city in Ethiopia, after Addis Ababa, Mekelle, Dire Dawa, and Nazareth [31].

Gondar has long struggled with water shortages, a challenge exacerbated by rising temperatures and a growing population. According to a 2014 household survey conducted by the Gondar City Municipality, which included 800 randomly selected households across the city, average water consumption was just 41 L per household per day [32]. With an average household size of four, this translates to about 10 L per person per day [33], significantly below the World Health Organization's (WHO) recommended range of 50–100 L per person per day [32]. In terms of electricity, Gondar benefits from 24-h power service, although frequent power outages occur due to the limitations of the city's network infrastructure. The 2014 survey indicated that 70% of households had private meters, while 9.1% shared meters [32].

Strategically located at the crossroads of the Ethio-Sudan highway, Gondar has seen rapid growth in industry and construction. The city is expanding in all directions, driven by an influx of people from surrounding rural areas and satellite towns in search of better opportunities. For many, Gondar represents a place where their futures are shaped, both economically and socially. The city also draws substantial tourism due to its historical and cultural sites, such as the Church of Kusquam, the castles of Emperor Fasilides, and the famous Debre Berhan Selassie Monastery. Every year, hundreds of visitors come to explore these landmarks, contributing to the city's revenue and supporting local businesses. Additionally, Gondar is a gateway for tourists heading to the natural wonders of the Simien Mountains [31].

### 2.2. Study Design

The study employed a case study research design. Quantitative data were collected from a sample of households in three kebeles: Kebele 11/12 (Abajalle subcity), Kebele 09 (Arada subcity), and Kebele 04/05 (Adebabay Eyesus subcity). These kebeles were selected from a total of 29,438 households, the sampling frame, using simple random sampling. A total of 132 sample households were proportionally selected from each kebele using the Taro Yamane sample size determination formula (Equation (1)). However, due to issues such as redundancy, enumerator errors, sampling bias, and respondent mistakes, only 200 completed questionnaires were analyzed.

(1) $$\begin{array}{c} n=\frac{N}{1+N\;{\left(e\right)}^{2}} \end{array}$$

where *n* is the sample size, *N* is the total number of households (sampling frame), and *e* = 0.05.

Qualitative data were gathered from 17 focus group participants and key informants, selected purposively to represent Ethiopian Electric Utility (EEU), Water Supply and Sewerage Authority (WSSA), local kebele administrations, and community members. Secondary data were sourced from the Amhara Region Electric Utility, Gondar City Water and Sewerage office, UN population prospects, Gondar City administration, and satellite images from the United States Geological Survey (USGS). Land surface temperature (LST), normalized difference building index (NDBI), and normalized difference vegetation index (NDVI) data were extracted from Landsat 7 and 8 images. Primary data were collected through questionnaires, focus group discussions, and key informant interviews, while secondary data were obtained through note-taking, document review, and online searches [34].

### 2.3. Data Analysis

The satellite data used in this study comprised 13 cloud-free (< 10%) Landsat 8 Operational Land Imager (OLI)/Thermal Infrared Sensor (TIRS) and Landsat 7 images from 2008 to 2020 (Path 170, Row 51), sourced from the US Geological Survey website for the study area. All images were acquired during the dry season, as Landsat 8 and Landsat 7 OLI/TIRS data are more reliable in this period. The dry season is preferred for LST studies in tropical cities due to fewer cloud-cover issues, which can otherwise hinder accurate measurements [35]. Previous research has highlighted that the UHI effect is more pronounced during the dry season [36]. Additionally, this period was selected to avoid the influence of temporary green spaces that emerge only during the wet season [37]. Studies have also suggested that the greater availability of sunshine during the dry season is a crucial factor for reliable LST assessment [38]. The preprocessing of the satellite images involved radiometric calibration, atmospheric correction through dark-object subtraction, and destripping, using ArcGIS 10.3. The aim of these procedures was to convert the digital number (DN) values of the thermal bands (Bands 6, 10, and 11) into at-satellite brightness temperatures (TB) expressed in Kelvin [36]. After preprocessing, the LST was extracted, with emissivity calculated based on the proportion of vegetation, as outlined in Equation (2).

(2) $$\begin{array}{c} \text{LST}\;\left({\text{⁣}}^{{}^{\circ}}\text{C}\right)=\frac{\text{TB}}{1+\left(\lambda \text{ x }\left(\text{TB}/\rho \right)\ln\left(\varepsilon \right)\right.} \end{array}$$

where TB is the brightness temperature, *λ* is the wavelength for Landsat 8 Band 10 and Landsat 7 Band 6, *ρ* = 1438, and *ε* is the emissivity.

The rural LST was extracted after delimiting a buffer zone separating the city from the surrounding rural area, which is part of the town's ecological footprint, having distinct climate, vegetation, and topographic characteristics that distinguish it from the city. Using an identifier tool of ArcGIS 10.3, pixels with high and low LSTs were identified. The average urban and rural LST was used to compute the SUHII of the study area using the model (Equation (3)).

(3) $$\begin{array}{c} \Delta T=T_{U}-T_{R} \end{array}$$

where Λ*T* is the change in temperature (SUHII), *T*_*U*_ is the average urban temperature, and *T*_*R*_ is the average rural temperature.

#### 2.3.1. The Path Model

Path modeling is a part of structural equation modeling (SEM). It is frequently used to study patterns of causality among a set of variables [39]. It allows users to investigate ways of effect within a system of variables and the impact of a group of predictor variables on multiple dependent variables.

The path model developed for this study is designed to examine the complex causal pathways among some of the drivers of heat island effects and their direct and indirect impacts on water and electricity consumption. The model can be estimated as a series of regression analyses with each variable defined in logical prior variations. The electricity (kilowatt-hour) and water consumption (cubic meter) were calculated using Equations (4) and (5), respectively.

(4) $$\begin{array}{c} \text{Econ}=\text{BPs}+\text{BTem}+\text{BImps}+e \end{array}$$

(5) $$\begin{array}{c} \text{Wcon}=\text{BPs}+\text{BTemp}+\text{BVg}+\text{BLs}+e \end{array}$$

where Temp is the SUHII, Ls is the average lot/residential land/size, Imp is the proportion of impervious surface (NDBI), Veg is the NDVI value, Econ is the per capita electricity consumption, Wcon is the per capita water consumption, Ps is the sample population, *B* is the beta (*β*) coefficient, and *e* is the error coefficient.

The inputs NDVI and proportion of impervious surfaces (NDBI) were derived using the surface reflectance of bands (red) and near infrared (NIR) for NDVI and middle infrared (MIR) and NIR for NDBI of Landsat 7 and 8, as shown in Equations (6) and (7), respectively.

(6) $$\begin{array}{c} \text{NDVI}=\frac{\rho \left(\text{Red}\right)-\rho \left(\text{NIR}\right)}{\rho \left(\text{Red}\right)+\rho \left(\text{NIR}\right)} \end{array}$$

(7) $$\begin{array}{c} \text{NDBI}=\frac{\rho \left(\text{MIR}\right)-\rho \left(\text{NIR}\right)}{\rho \left(\text{MIR}\right)+\rho \left(\text{NIR}\right)} \end{array}$$

##### 2.3.1.1. Variable Identification for the Path Model

**Figure 3:**
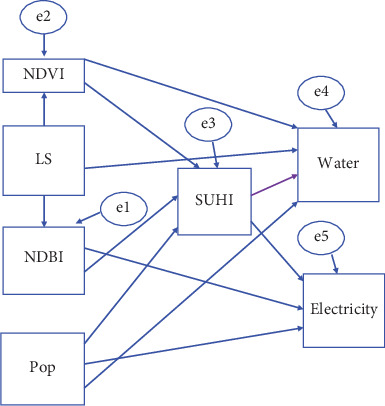
The path model.

In constructing the path model for impact analysis, the variables that may contribute to single-family residential water use and residential electricity consumption are identified. These variables are mean residential land use, population size, vegetation cover (NDVI), the proportion of impervious surfaces (NDBI), and SUHII. These variables were identified using previously reported procedures [23], preliminary surveys and interviews, and observing the existing situation. As indicated in Figure 3, the independent exogenous variables are lot size (LS) and population size. In contrast, the dependent endogenous variables are electricity consumption, water consumption, NDVI, NDBI, SUHII, and error variables.

The variables used were with different units of measurement. These have been normalized using the following formula (Equation (8)).

(8) $$\begin{array}{c} I=\frac{T_{w}-T_{i}}{T_{m}-T_{i}} \end{array}$$

where *I* is the variable to be normalized, *T*_*w*_ is the actual value of the variable, *T*_*i*_ is the minimum value of the variable to be normalized, and *T*_*m*_ is the maximum value of the variable to be normalized.

**Figure 4:**
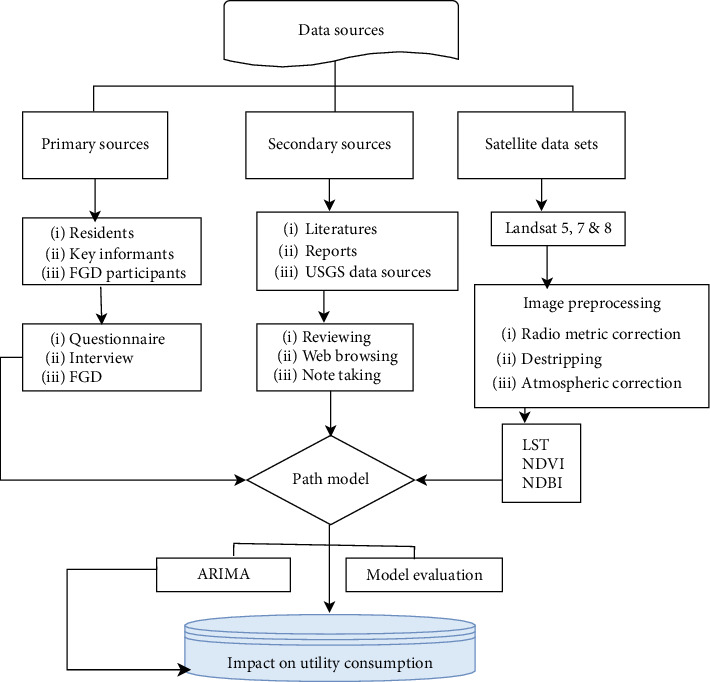
Methodological framework of the study.

As indicated in Figure 4 data from the satellite images of the USGS, UN population prospect, and Gondar City administration were computed using APSS-AMOS (Statistical Package for Social Sciences-Analysis of Moment Structures) and XLSTAT and analyzed using the path model, principal component analysis (PCA), and multiple linear regression (MLR). In contrast, data from sample respondents were analyzed using the Likert scale. The *β* coefficient, the correlation matrix, the sum of the squares, and the mean value of the Likert scale were used to determine the factor with a severe impact on per capita water and electricity consumption. Future consumption pattern was traced using autoregressive integrated moving average (ARIMA). The data from key informants and group discussants were analyzed thematically in line with the other measurement results. Using the above models (the path and ARIMA), the impact SUHII has on water and electricity consumption was analyzed.

## 3. Results

### 3.1. The Path Model Evaluation

#### 3.1.1. Multicollinearity

A screening of bivariate correlations will be done to look for multicollinearity. Bivariate correlations above *r* = 0.85 may be a warning of trouble. One of the redundant variables will be eliminated if that takes place in the model. All correlation coefficients in the model are less than 0.85.

#### 3.1.2. Outliers

It was examined for univariate and multivariate outliers after the model had run. The existence of an extreme variable(s) or two or more extreme scores on several different variables (multivariate outliers) was examined. Cochran's test (*p* > 0.05) revealed that there are no outliers in the dataset.

#### 3.1.3. Normality

A multivariate distribution is presumed to be normal by the majority of SEM statistics. It can be troublesome to violate this condition since non-normality will reduce the precision of statistical analyses. It is impractical to test whether the multivariate normality assumptions are true since doing so would require looking at an unlimited number of linear combinations. Examining each independent variable's distribution is one approach. As a result, checking for univariate normality can help identify any potential problems with multivariate normality. The researcher checked each observed variable's distribution for skewness and kurtosis to see if univariate normality existed. Absolute values greater than 3.0 are considered excessive for the skewness index. However, as shown in Table 1, estimated values for skewness for electricity and water are both |−2.534| and |−0.412|, respectively. Both of their distributions have a negative skew. Absolute values for the kurtosis index greater than 10.0 indicate a problem, and values greater than 20.0 are extreme. Water is platy kurtic, while electricity is lepto kurtic, or flat, as shown by the estimated value for kurtosis for water (Table 1), which is |−1.295|, and electricity (Table 1), which is |5.525|.

#### 3.1.4. Model Fit Indices

i. *Absolute fit indices (GFI (the goodness-of-fit index) and χ*^2^* (chi-square))*: absolute fit indices were calculated to evaluate how well a model fits the observable data. The variance accounted for by the entire model is what is meant by GFI, which is comparable to *R*^2^, which is used in regression to summarize the variation explained in a dependent variable. A significant *χ*^2^ implies that the model does not well match the sample data, while a nonsignificant *χ*^2^ shows that the model does. As shown in Table 2, the computed *p* value is 0.314 (*p* > 0.01), which is insignificant, and the model's *χ*^2^ result is 9.344, the minimum value. The variance explained by the dependent model is calculated to be 84.5% (0.845) (Table 2), or the GFI value, which is comparable to *R*^2^, explains the factors that are taken into account for the complete model.
ii. *Comparative fit index (CFI)*: this index was used to assess how well the researcher's model fits the data compared to a more constrained model known as the independence or null model, which assumes no correlations between the variables. As observed in Table 2, CFI has a range of 0–1.0, with values nearer 1.0 denoting a better match. The calculated CFI value, which is 0.936, is closer to one that indicates a better fit (Table 2).
iii. *Root mean square error approximation (RMSEA)*: the value of 0.00 for the RMSEA indicates that the model perfectly matches the data. As shown in Table 2, the model's computed RMSEA is 0.118, which is close to zero and indicates that the model is fit.
iv. *Root mean square residual (SRMR)*: based on covariance residuals, the SRMR index uses smaller values to indicate a better fit. How big of a difference there is between the observed data and the model is summarized by the SRMR. In this regard, a model that produces an SRMR of 0.004 denotes a perfect fit (Table 2). For an acceptable fit, the model typically needs to provide an SRMR of less than 0.10.

#### 3.1.5. Model Identification Procedures for the Path Model

One method of determining whether a model is just, over, or underidentified is by looking at the degrees of freedom. Subtracting the number of distinct sample moments from the total number of parameters that need to be estimated yields the degrees of freedom for a model. There are 28 separate sample moments, 20 distinct parameters that must be evaluated, and eight degrees of freedom, or the difference between the two. The model is said to be just identified if the degree of freedom is equal to zero; it is said to be overidentified if the degree of freedom is greater than zero. If the degree of freedom is lower than zero, the model is underidentified. Additionally, overidentified models effectively examine the connections between the variables and their effects. The path model is too identifiable in the instance being studied.

#### 3.1.6. Variable Identification Procedures for the ARIMA Model

Electricity and water consumption depend on economic, demographic, and climatic conditions. Population, SUHII, the proportion of impervious surfaces, mean LS, and vegetation cover are identified as variables impacting water and electricity consumption. Among these, the variable with the most significant influence was selected for the projection. The PCA correlation matrix, the MLR Type III sum of squares, and the mean value of the Likert scale were used in the identification process. The *β* value of the path model, the PCA correlation matrix, the sum of squares of the MLR, and the mean value of the Likert scale were used to identify the influential variable and make the projection in line with the distribution pattern of this variable.

The PCA correlation matrix in Table 3 indicates a significant correlation between the independent and dependent variables. The correlation matrix shows that SUHII has a more meaningful (*p* < 0.05) positive association with water and electricity than the other variables. That is, an increase in SUHII brings a relative increase in water and electricity consumption and vice versa. It also tells the influence SUHII has on water and electricity consumption.

The MLR model in Table 4 indicates the variables' influence on water and electricity consumption. The independent variables explain the variability of the dependent variables (water and electricity). But, based on the Type III sum of squares, SUHII is more influential than other variables.

Table 5 is a Likert scale table. Data obtained from the sample respondents using a questionnaire were analyzed using mean scores calculated for each statement in the Likert scale using Equations (9) and (10), respectively:

(9) $$\begin{array}{c} \text{Mean score}=\Sigma \frac{\left(f_{i}\times \text{Likert item score}\right)}{\text{Number of respondents}} \end{array}$$

(10) $$\begin{array}{c} \text{Number of respondents}=\frac{\text{Percentage share of each category}\times \text{Total number of respondents}}{100} \end{array}$$

The range of interpreting the Likert scale mean score was given as 1.0–2.4 (negative attitude), 2.5–3.4 (neutral attitude), and 3.5–5.0 (positive attitude). According to this study, the mean values indicate the impact's severity; hence, the higher the value, the higher the degree of the effect. The result of the Likert scale computation (Table 5) indicates the impact SUHI has on per capita water and electricity consumption. It is one of the highest for water (4.495). This is probably due to the utilization of more water for bathing and drinking because of the intensified heat intensity. In addition, the per capita electricity consumption, as indicated in Table 5, is also higher (4.16) due to the utilization of electric gadgets (coolants), which consume more power.

The key informants and group discussants also indicated that the impact of SUHI on water and electricity consumption is becoming stronger. Current water utilization is higher for drinking, bathing, or vegetation growth. Similarly, electricity consumption for ventilation and refrigeration is higher due to the intensified heat.

The path model, the PCA, MLR, and the Likert scale indicated the more significant influence SUHII has on water and electricity consumption. Therefore, it is chosen to project future consumption patterns in line with its future way of distribution. The model selected for projection is an ARIMA, as reported by previous research [40].

#### 3.1.7. Model Identification Procedures for the ARIMA

The model identification step's goal is also to choose values for *d*, then *p*, and finally *q* in the ARIMA (*p*, *d*, *q*) model. It will be determined to be a reasonable model for the data by taking into account the patterns of autocorrelations and partial autocorrelations. The values of *d* (differencing), *p* (autoregressive (AR) order), and *q* (moving average (MA) order) are determined during the identification phase. The first of the three processes included in ARIMA models is autoregression (*p*). In an AR process, a forecast is made based on prior time series observations. The differencing (*d*) or integration component of an ARIMA model tries to make a series stationary. The MA component of an ARIMA model tries to predict future values of the series based on deviations (errors) from the series mean observed for previous values. In an MA process, each value is determined by the weighted average of the current disturbance and one or more previous disturbances. The order of the MA process specifies how many previous disturbances are averaged into the new value [41].

The models were taken into consideration with p and q ranging from 1 to 3. The models that were examined included (1, 1, 1), (1, 1, 2), (1, 1, 3), (2, 1, 1), (2, 1, 2), (3, 1, 1), and (3, 1, 2). As the best model for the research area, the model with the lowest archaic information criterion corrected (AICC) value was chosen. The model for water, SUHII, and electricity was composed of the numbers (3, 1, 2), (3, 1, 1), and (1, 1, 2), respectively.

### 3.2. Impact Analysis

Using the *β* value of SUHII, taking other factors constant, the study determined that the study area's per capita water consumption was 2.378 m^3^/1°C/annum, or 6.5 L/day, significantly less than the WHO requirement (Table 6). Although the demand for water for cleaning, vegetation growth, and bathing has likely increased along with population expansion and heat waves, actual water consumption has been shown to be less than the municipality's 2014 forecast despite these factors. The number of the population, the amount of vegetation, and the size of the compounds are among the factors that are taken into account for impact analyses on water usage. In comparison to SUHII, their increase or decrease is also of utmost relevance, though to a lesser extent (Table 6).

The formula for computing water consumption is shown in Equation (11).

(11) $$\begin{array}{c} W_{c}=0.678+0.5X_{1}+0.2X_{2}+0.4X_{3}+0.6X_{4}+1.1 \end{array}$$

Similarly, using the *β* value of SUHII, the study determined that per capita electricity consumption is 1.616 kW/1°C/annum, equivalent to 4.4 W/day, taking other factors constant (Table 6). Similar to water consumption and demand, the rise in heat intensity and the increase in population raised the demand for electricity due to the consumers' unlimited need for electricity for refrigeration and, to some extent, ventilation. Actual consumption is below the 2013 standard due to the low provision of electricity by the supplier. From the considered variables for impact analysis (Table 7), impervious surface coverage has an effect explained by its influence on SUHII. Population and electricity consumption are closer to growth in SUHII. The formula for computing electricity consumption is shown in Equation (12).

(12) $$\begin{array}{c} E_{c}=0.116+0.6X_{1}+0.2X_{2}+0.3X_{3}+0.9 \end{array}$$

Equations (9) and (10) were validated by checking the statistical significance (*p* value). As it is observed in Table 6, the *p* value is statistically significant (*p* < 0.05). The other validation technique used was checking the assumptions of the model. This was done by linearity and multicollinearity tests. Linearity was checked by testing for skewness and kurtosis, and hence, the data was found to be normally distributed. The bivariate correlation test indicated that multicollinearity was not a problem. The other validation technique used was the model's performance metrics. The Akaike information criterion (AIC) value was found to be significant for the model.

SUHII is affected by vegetation cover, impervious surfaces, and population size (Table 7). However, the proportion of impervious surfaces has a superior impact than the other two. On the other hand, LS affects the ratio of impervious surfaces and vegetation. Its increment or reduction has a concomitant effect on the size of buildings and the amount of vegetation cover.

### 3.3. Projecting Urban Utility Consumption In Line With SUHII

**Figure 5:**
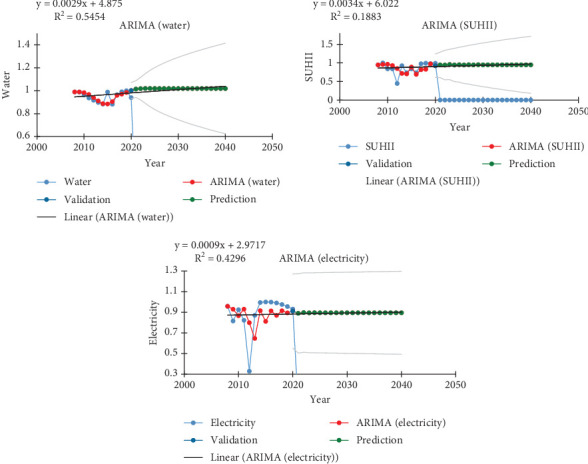
Observed, synthetic, and forecast of water (cubic meters), SUHII (degrees Celsius), and electricity (kilowatts) consumption.

The data was made stationary before being made accessible for projection and going through the ARIMA procedures. By contrasting the observed series with the forecast series produced by the ARIMA model, the model's accuracy was evaluated. A 95% confidence interval was created for a forecast series with a 16-year lead time.  Figure 5 displays the observed and anticipated SUHII, water, and power consumption series for the research area chosen for this investigation. The forecast data series was created after the observed dataset. Future SUHII, water, and energy use are all expected to rise. The increase is 0.0034°C, 0.0029 m^3^, and 0.0009 kW in magnitude, respectively.

**Figure 6:**
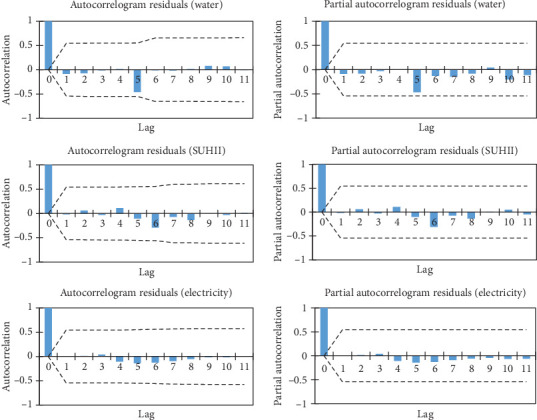
Autocorrelation and partial autocorrelation function of water (cubic meters), SUHII (degrees Celsius), and electricity (kilowatts).

The autocorrelation function (ACF) and partial autocorrelation function (PACF) residual plots were investigated after the models had been fitted [40]. The residuals were found to be within the confidence intervals, demonstrating the model's suitability and good fit. That is an analysis of a correlogram of an ACF and PACF graphic. The residuals that remain after the model has been fitted must meet the criteria of a white noise process in order to be considered a successful forecasting model. The correlogram of the ACF and PACF of the residuals of the SUHII, water, and electricity data was clearly within the confidence interval, as shown in Figure 6. This demonstrated their significance and the independence of the residuals, satisfying the residual requirement. Additionally, there were no trends found in the residuals, further proving that the models could be utilized to represent the observed data. It denotes that there was no pattern or trend and that the residuals were small in size. The disparity between the actual and predicted data is known as the residual. The prediction value is implied to be as close as possible to the observed data, further demonstrating the model's performance effectiveness. Therefore, residuals are used to verify models.

For the SUHII (degrees Celsius), water (cubic meters), and electricity (kilowatt-hour) models, the performance effectiveness of the model was assessed using the sum of square error (SSE), mean absolute percentage error (MAPE), mean square error (MSE), and root mean square error (RMSE).  Table 8 displays the values for the models. The MAPE, an objective metric, was used in the table provided to assess the models' accuracy in making predictions. Its modest value serves as proof that the models are adequate.

#### 3.3.1. Correlations Between SUHII and Water and Electricity

**Figure 7:**
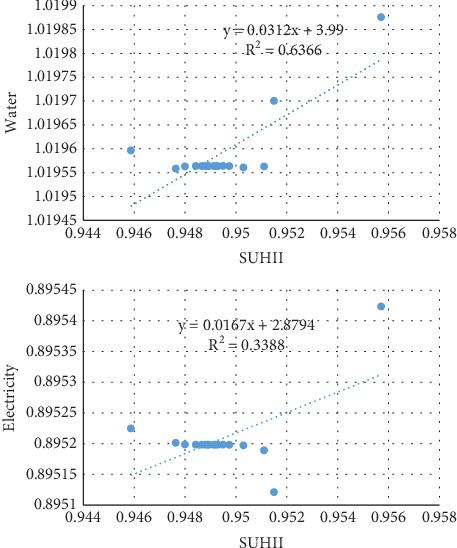
Correlation between SUHII (degrees Celsius) and water (cubic meters) and electricity (kilowatts) consumption.

The forecast of SUHII significantly correlates with water in cubic meters (*r* = 0.6) and electricity in kilowatts (*r* = 0.3). As indicated in Figure 7, the increase of SUHII by 1°C will increase water by a factor of 0.0312 m^3^ while it is 0.0167 kW for electricity. Sixty-four percent of the future water consumption variability depends on SUHII. In comparison, 33% of the future variability in electricity is accounted for by SUHII.

#### 3.3.2. The Forecast Models

(13) $$\begin{array}{c} Y_{t}=-0.450Y_{t-1}-0.057Y_{t-2}-0.249Y_{t-3}-0.132\in_{t-1}+4.112 \end{array}$$

where *Y*_*t*_ is the SUHII at time *t*, *Y*_*t*−1_ is the SUHII in the previous 1 year, *Y*_2−1_ is the SUHII in the previous 2 years, *Y*_3−1_ is the SUHII in the previous 3 years, and *Ε*_*t*−1_ is the SUHII residuals in the previous 1 year

(14) $$\begin{array}{c} Y_{t}=0.371Y_{t-1}-0.0237Y_{t-2}-0.031Y_{t-3}-1.125\in_{t-1}+1\in_{t-2}+2.250 \end{array}$$

where *Y*_*t*_ is the water consumption at time *t*, *Y*_*t*−1_ is the water consumption in the previous 1 year, *Y*_2−1_ is the water consumption in the previous 2 years, *Y*_3−1_ is the water in the previous 3 years, and *Ε*_*t*−1_ is the water consumption residuals in the previous 1 year

(15) $$\begin{array}{c} Y_{t}=-0.343\;Y_{t-1}-0.277\in_{t-1}-0.622\in_{t-2}+1.183 \end{array}$$

where *Y*_*t*_ is the electricity consumption at time *t*, *Y*_*t*−1_ is the electricity consumption in the previous 1 year, *Ε*_*t*−1_ is the electricity consumption residuals in the previous 1 year, and *E*_*t*−2_ is the electricity consumption residuals in the previous 2 years.

##### 3.3.2.1. Application of the Forecasted Model

The forecasted models for SUHII (degrees Celsius), water consumption (cubic meters), and electricity consumption (kilowatts) of the study area are presented in Equations (11), (12), and (15), respectively, using the values in Table 9. Forecasting water and energy consumption in line with the changing pattern of SUHII is vital for water resource planning and management and energy load forecasting. It is also essential to avert energy and water crises by helping to balance the supply and demand of these utilities by considering thermal changes. Water and electricity consumption forecasting is becoming an essential tool for the design, operation, and management of supply systems in activities such as planning new developments or system expansion; estimating the size and function of reservoirs and substations, pumping stations, and pipe capacities; and for urban electricity and water management issues (pricing policies and consumption restrictions) [17]. It is also very vital for better informed management and decisions and an assessment of additional requirements to balance supply and demand [17].

## 4. Discussion

By demonstrating an increase in surface air temperature, global and regional models and observations have shown that the climate of Earth is warming. By examining the temporal increase in air temperature caused by greenhouse gas (GHG) emissions in the present and the future, the Intergovernmental Panel on Climate Change (IPCC) bolstered this argument [42]. Research that shows an increase in the world's mean surface temperature since 1990 has further corroborated and enhanced this [42]. Ethiopia's average surface temperature has climbed in recent decades after rising significantly in the previous 40–50 years. Contrarily, Teshome et al. [43] and Ayalew et al. [44] suggested that the country's temperature will continue to climb in the future.

According to Watkins, Palmer, and Kolokotroni, cities and other metropolitan regions would likely experience the worst effects of this temperature change [45]. Heat-related illnesses such as heat stress, cramps, syncope, edema, exhaustion, and stroke are global threats to human health that are brought on by exposure to hot weather [46]. Urban utility demand and consumption are likewise affected [23]. According to the same study, consumption in Arizona was 4.61 KL/1°C/year/person in 2006 [23]. According to Ali and Terfa, the East Wollega Zone of Ethiopia's population used 15–26 L of water per person per day [47]. According to Arsiso et al. [48], this amount was 110 L/capita/day in 2012 in Addis Ababa but 135 L/capita/day in 2020. In Gondar in 2014, it was only 14 L/day, which is less than the WHO-recommended range of 50–100 L/day. This study determined that the study area's per capita water consumption was 2.378 m^3^/1°C/annum, or 6.5 L/day, significantly less than the WHO requirement (Table 6, Section 3.1).

Elevated temperatures in cities also increase energy consumption and demand for cooling systems, lights, and appliances by 1.5%–2.0% for every 1°F (0.6°C) [49]. This figure rose to 1.5%–3.1% for every 1.74°C–3.43°C in Thailand [50] and to 3%–6% for every 1°C–1.4°C in the United States [51]. There is also a growing demand for electricity in Ethiopia, about 25% per year [24]. The same study indicated the electricity consumption per capita in Ethiopia in 2013 was 65 kW/annum, which is 17.8 W/day [24]. This study also indicated that per capita electricity consumption is 1.616 kW/1°C/annum, equivalent to 4.4 W/day, taking other factors constant (Table 6, Section 3.1).

According to studies, rising temperatures would cause a spike in electricity demand and consumption in the future. According to the study conducted in Thailand, a rise in the country's mean annual temperature of 1.74°C–3.43°C will result in an increase in peak electricity demand and consumption of 3.7%–8.3% in the 2050s and 6.6%–15.3% in the 2080s [50]. With an increase of 3.7°C from the baseline in 2055, this percentage will climb to 13%–20% in the United States [51]. In Ethiopia, there will be an increase in demand for both water and energy in the coming years due to climate change and population growth [48] in addition to economic expansion [25]. This predicted growth in consumption in accordance with SUHII in the study area was determined by the ARIMA projection. The amount of water consumed in 2040 will be 4 m^3^/1°C/annum, or 11 L/day, while the amount of energy consumed will be 2.9 kW/1°C/annum, or 8 W/day. The WHO requirement as well as Ethiopia's 2013 estimate for energy use will not be met after 16 years. It is clear from the correlation study (Figure 7) that providers should also take other factors into account in addition to SUHII when making their plans.

## 5. Conclusion

Surface UHIs significantly impact residential water use and electricity consumption. Studies indicate that it has a considerable influence on such urban utilities. Therefore, studying the impact and projecting the future is paramount for policy development and planning. This study is aimed at analyzing the effects of SUHI (degrees Celsius) urban heating on water (cubic meters) and electricity consumption (kilowatts). The result of the study indicated that the historical SUHII (degrees Celsius) data positively correlates (*r* = 0.5 and *r* = 0.6) with per capita water (cubic meters) and electricity consumption (kilowatt-hour). The study also ascertained the per capita water consumption in the study area to be 2.378 m^3^/1°C/annum, equivalent to 6.5 L/day. Per capita, electricity consumption is 1.616 kW/1°C/annum, equal to 4.4 W/day, taking other factors constant.

On the other hand, the ARIMA model suggests a future increase in per capita water (cubic meters) and electricity consumption (kilowatts-hour) due to the rise in SUHII (degrees Celsius). From the study's findings, it can be concluded that SUHII has a significant positive association with per capita water and electricity consumption. Similarly, it significantly impacts per capita water and electricity consumption. Therefore, the electric utility needs to incorporate the effects of thermal change within its load forecasting and system planning regime. Also, WSSA needs to assess the risk of thermal change on water availability and actual consumption. They should also make accurate demand forecasts, which coincide with the changing urban climate. In order to optimize electricity consumption, it is better to enhance the capacity of the power system to operate under a range of future environmental and socioeconomic conditions. In addition to this, increasing photovoltaic (PV) systems reduce the effects of peak demand since this energy source closely matches the diurnal demand for electricity. Furthermore, energy efficiency and demand response targets that provide substantial cushioning against the effects of higher temperatures should be in place. With respect to water consumption, investment policies that can address private developers in the sector by way of providing incentives and liberalizing markets should be in place. In addition to this, granting residents freedom so that they recognize their necessity and hence, change their perception, habits, and behavior of consumption with the changing environment. Furthermore, subsidizing the sector to prioritize accessibility in delivering water services. Last but not least, supporting the management of water reservoirs with the use of modern possible seasonal and short-term hydrologic forecasts and numerical decision support tools. Finally, enhancing the awareness of the residents and other stakeholders about the effects of UHI on water so that they can recycle and reuse wastewater.

## Figures and Tables

**Table 1:** Normality test results.

**Assessment of normality**	**Variables**						
	**POP**	**LS**	**NDBI**	**NDVI**	**SUHII**	**Electricity**	**Water**
Skew	−0.144	1.271	1.726	−0.309	−1.525	−2.534	−0.412
Kurtosis	−1.192	0.582	1.981	−0.806	1.843	5.525	−1.295

**Table 2:** Model fit indices.

**Indices**	**Model fit indices**						
	**SRMR**	**GFI**	**RMSEA**	**CFI**	**AIC**	**χ** ^2^	**p** ** value**
Value	0.004	0.845	0.118	0.936	49.344	9.344	0.314

**Table 3:** Correlation matrix at 95% confidence level (PCA).

**Variables**	**UHI**	**NDVI**	**NDBI**	**POP**	**LS**	**Water**	**Electricity**
SUHII	1	−0.373	0.233	0.362	0.027	0.579	0.691
NDVI	−0.373	1	0.169	−0.647	0.291	0.097	0.309
NDBI	0.233	0.169	1	−0.214	0.671	0.319	0.121
POP	0.362	−0.647	−0.214	1	−0.469	0.190	0.438
LS	0.027	0.291	0.671	−0.469	1	0.166	0.122
Water	0.579	0.097	0.319	0.190	0.166	1	0.198
Electricity	0.691	−0.309	0.121	0.438	0.122	0.198	1

**Table 4:** Type III sum of the squares (MLR).

**Electricity**					**Water**			
**Source**	**Sum**	**Mean**	**F**	**Pr** > **F**	**Sum**	**Mean**	**F**	**Pr** > **F**
UHI	0.054	0.054	6.301	0.066	0.097	0.097	75.216	0.001
NDVI	0.001	0.001	0.085	0.785	0.000	0.000	0.287	0.620
NDBI	0.022	0.022	2.557	0.185	0.002	0.002	1.732	0.258
POP	0.043	0.043	5.028	0.088	0.001	0.001	1.012	0.371
LS	0.039	0.039	4.611	0.098	0.002	0.002	1.769	0.254

**Table 5:** Likert scale.

	**Major impacts**	**Categories percentage values**					**Mean**
		**Strongly agree**	**Agree**	**Neutral**	**Disagree**	**Strongly disagree**	
Impacts of SUHII	Water consumption	65	16.5	9	5	4.5	4.495
	Electricity consumption	58.5	18.5	9.5	7.5	6	4.16
	Severe health problem	56.5	15.5	12	10	6	4.065
	Severe ecological problem	57	17.5	9	10	6.5	4.085
	Problem in vegetation growth	57	16.5	11.5	9.5	5.5	4.1
	Problem in air quality	57	14.5	11.5	10	7	4.045
	Problem on climate	56.5	16.5	11.5	10.5	5	4.105

**Table 6:** Impact of SUHII on water and electricity consumption.

**Variable**		**Intercept**	**B**	**SE**	**p**	**Variable**		**Interest**	**B**	**SE**	**p**
**Endogenous**	**Exogenous**										
						**Endogenous**	**Exogenous**				
Water (m^3^)	NDVI	0.678	0.5	0.5	*p* ≤ 0.01	Electricity (kW)	SUHII	0.116	0.6	0.3	*p* ≤ 0.01
	LS		0.2	0.4	*p* ≤ 0.05		POP		0.2	0.2	*p* ≤ 0.05
	POP		0.4	0.1	*p* ≤ 0.01		NDBI		0.3	0.4	*p* ≤ 0.01
	SUHII		0.6	0.1	*p* ≤ 0.01						

*Note:B* = beta coefficient, *p* = significance level.

Abbreviation: SE = standard error.

**Table 7:** The impacts of NDVI, NDBI, POP, and LS.

**Variable**		**Intercept**	**B**	**SE**	**p**	**Variable**		**Interest**	**B**	**SE**	**p**
**Endogenous**	**Exogenous**					**Endogenous**	**Exogenous**				
SUHII (°C)	NDVI	0.547	−0.2	0.2	*p* ≤ 0.01	NDBI	LS	0.33	0.29	0.1	*p* ≤ 0.01
	NDBI		0.5	0.4	*p* ≤ 0.05	NDVI	LS	0.27	0.67	0.27	*p* ≤ 0.01
	POP		0.2	0.3	*p* ≤ 0.01						

*Note:B* = beta coefficient, *p* = significance level.

Abbreviation: SE = standard error.

**Table 8:** Goodness-of-fit statistics (ARIMA).

**Goodness-of-fit statistics**	**Variables**		
	**Water**	**Electricity**	**SUHII**
SSE	0.01427	0.373911	0.286999
MSE	0.001297	0.033992	0.026091
RMSE	0.036017	0.184369	0.161526
MAPE	2.284011	21.99135	15.88722

**Table 9:** ARIMA and parameters.

**Model**	**Parameter**	**Estimate coefficient**	**Hessian standard error**
ARIMA (3, 1, 1) (SUHII in °C)	Φ_1_	−0.450	1.418
	Φ_2_	−0.057	0.872
	Φ_3_	−0.249	0.301
	Θ_1_	−0.132	1.522

ARIMA (3, 1, 2) (water in m^3^)	Φ_1_	0.371	0.470
	Φ_2_	−0.023	0.416
	Φ_3_	−0.031	0.377
	Θ_1_	−1.125	0.477
	Θ_1_	1.000	0.510

ARIMA (1, 1, 2) (electricity in kW)	Φ_1_	−0.343	1.134
	Θ_1_	−0.277	1.776
	Θ_1_	−0.622	0.640

*Note:*Φ = autoregressive variable, Θ = moving average variable.

## Data Availability

The Landsat satellite images analyzed during the study are collected from the US Geological Survey. These datasets were derived from the following public domain resources: https://usgs.gov/.
